# Analysis of banana transcriptome and global gene expression profiles in banana roots in response to infection by race 1 and tropical race 4 of *Fusarium oxysporum* f. sp. *cubense*

**DOI:** 10.1186/1471-2164-14-851

**Published:** 2013-12-05

**Authors:** Chunqiang Li, Jiaofang Shao, Yejun Wang, Wenbin Li, Dianjing Guo, Bin Yan, Yiji Xia, Ming Peng

**Affiliations:** Institute of Tropical Bioscience and Biotechnology, Chinese Academy of Tropical Agricultural Sciences, Haikou, China; Hainan University, Haikou, China; Department of Biology, Hong Kong Baptist University, Hong Kong, China; School of Life Science Chinese University of Hong Kong, Hong Kong, China; Partner State Key Laboratory of Agrobiotechnology, Chinese University of Hong Kong, Hong Kong, China

## Abstract

**Background:**

Cavendish, the most widely grown banana cultivar, is relatively resistant to Race 1 of *Fusarium oxysporum* f. sp. *cubense* (Foc1) which caused widespread Panama disease during the first half of the 20^th^ century but is susceptible to Tropical Race 4 of Foc (Foc TR4) which is threatening world banana production. The genome of the diploid species *Musa acuminata* which is the ancestor of a majority of triploid banana cultivars has recently been sequenced. Availability of banana transcriptomes will be highly useful for improving banana genome annotation and for biological research. The knowledge of global gene expression patterns influenced by infection of different Foc races will help to understand the host responses to the infection.

**Results:**

RNA samples from different organs of the Cavendish cultivar were pooled for deep sequencing using the Illumina technology. Analysis of the banana transcriptome led to identification of over 842 genes that were not annotated by the *Musa* genome project. A large number of simple nucleotide polymorphisms (SNPs) and short insertions and deletion (indels) were identified from the transcriptome data. GFP-expressing Foc1 and Foc TR4 were used to monitor the infection process. Both Foc1 and Foc TR4 were found to be able to invade banana roots and spread to root vascular tissues in the first two days following inoculation. Digital gene expression (DGE) profiling analysis reveal that the infection by Foc1 and Foc TR4 caused very similar changes in the global gene expression profiles in the banana roots during the first two days of infection. The Foc infection led to induction of many well-known defense-related genes. Two genes encoding the ethylene biosynthetic enzyme ACC oxidase and several ethylene-responsive transcription factors (ERF) were among the strongly induced genes by both Foc1 and Foc TR4.

**Conclusions:**

Both Foc1 and Foc TR4 are able to spread into the vascular system of banana roots during the early infection process and their infection led to similar gene expression profiles in banana roots. The transcriptome profiling analysis indicates that the ethylene synthetic and signalling pathways were activated in response to the Foc infection.

**Electronic supplementary material:**

The online version of this article (doi:10.1186/1471-2164-14-851) contains supplementary material, which is available to authorized users.

## Background

Banana (*Musa* spp.) is grown in tropical and sub-tropical areas and is the fourth most important crop in developing countries behind rice, wheat, and corn [1]. In many countries in Africa and Asia, it is a major staple food crop. There are two types of banana crops: sweet “desert” banana and starchier “cooking” banana which is also called plantains. More than 100 million tons of banana and plantain were produced worldwide in 2010 according to the FAO estimates (http://faostat.fao.org/site/339/default.aspx).

Bananas are tall monocotyledonous plants. A large majority of cultivated sweet bananas and cooking bananas are seedless triploid varieties (2n = 3x = 33) that were derived from intra- or inter-specific crosses between two diploid wild species, *M. acuminata* (whose genome is designated ‘AA’) and *M. balbisiana* (whose genome is designated ‘BB’) [1–3]. The most common varieties of sweet bananas are the triploid type with the AAA genome derived from crosses within *M. acuminata*, while the most common cooking bananas (the AAB or the ABB genome type) were the triploid originated from crosses between *M. acuminata* and *M. balbisiana*. Wild diploid banana produces seeds, whereas cultivated triploid banana is sterile but develops parthenocarpic fruits.

Before 1960s, the cultivar ‘Gros Michel’ was the principal sweet banana variety. However, a world-wide outbreak of banana wilt disease, which is also termed Panama disease caused by Race 1 of the fungal pathogen *Fusarium oxysporum* f. sp. *Cubense* (Foc), led to world-wide destruction of bananas in 1940s-1960s. The ‘Cavendish’ sub-group was later found to be more resistant to Foc Race 1 and replaced Gros Michel as the most widely distributed banana cultivars today. Cavendish is believed to be originated in southern China [3]. However virulent strains of Race 4, particularly Tropical Race 4 (Foc TR4), to which Cavendish is susceptible, have rapidly been spreading to banana production areas and has caused substantial losses in many countries in Asia and Australia [4]. As cultivated banana is generally asexually propagated using suckers from rhizomes, the large scale cultivation of the single genotype is particularly vulnerable to pathogens. The lack of banana diversity and varieties that are resistant to Foc TR4 and the difficulty in the banana breeding process have raised a serious concern that banana is again facing the threat of disappearing from the shops [5] (http://www.the-scientist.com/news/display/54710/).

The draft sequence of the 523-megabase genome of a doubled-haploid *Musa acuminata* genotype has recently become publically available [6]. The genome of cultivated banana are expected to be more complex due to its polyploidy and heterozygosity. Here we report analysis of the transcriptome from mixed tissues and organs of Cavendish plants obtained using the Illumina sequencing technology. The analysis led to identification of additional genes which were not predicted from the genome sequencing project. The differences in pathogenesis process of the different Foc races and host responses to their infection remains little known. We carried out digital gene expression profiling to compare global gene expression patterns in the roots of Cavendish plants infected with Foc1 and Foc TR4. Our study generated useful resources for the banana research community for understanding Foc-banana interactions.

## Results and discussion

### Analysis of the banana transcriptome and identification of genes that were not previously annotated in the *M. acuminata* genome

The RNA samples were isolated from various tissues of the Cavendish cultivar including leaves, pseudostems, roots, flowers, and developing fruits and were pooled and subjected to whole transcriptome shotgun sequencing (RNA-seq) using the Illumina’s HiSeq 2000 system. We sequenced two rounds (pair ends) of banana mRNA sequences and obtained a total of 26,666,670 reads and 2,400,000,300 nucleotides.

In total, 47411 different transcripts were identified through analysis of the sequence reads (Accession No. SRX317049) using TopHat [7] and Cufflinks [8], of which10545 transcripts map to the genes that were already annotated by *Musa* genome project [6]. The remaining 36866 transcripts found by Cufflinks analysis were further analysed. These potential novel transcripts were used as the queries in searching against the NCBI nr (non-redundant) database by BLASTx. In addition, the transcripts were also aligned to UniProt plant protein sequences (http://www.uniprot.org/uniprot/?query=taxonomy%3a33090&force=yes&format=fasta) [9] by BLASTx. The potential transcripts that are derived from more than one exon (based on their alignments with the *Musa* genome) or from a single exon but having a BLAST hit to known protein(s) at the cutoff E-value 1e-5 were considered to be more likely transcribed from genuine genes and are reported as novel banana transcripts in this study. Using this analysis, a total of 842 novel loci (which include 925 different transcripts as some loci produced alternative transcripts) were identified and listed in Additional file 1: Table S1. Additional file 1: Table S1 contains the sequences of the 842 transcripts, the predicted open reading frames (ORFs) and their translated peptide sequences, the locations of these novel genes in the *Musa* genome, and their relative transcript abundances (FPKM, Reads Per Kilobase of transcript per Million mapped reads) which were based on the numbers of their hits by RNA-seq and calculated by Cufflinks. These novel transcripts are designated by a number proceeded with ‘CUFF’ in Additional file 1: Table S1.

Among the novel transcripts identified from the analysis, many share a high sequence similarity to proteins encoded by other plant and/or non-plant genomes. Therefore, they are not truly novel genes but were not predicted or annotated through the *Musa* genome project. For instance, CUFF.40341 encodes an acidic endochitinase which has the highest FPKM (5154.29) among the ‘novel’ transcripts. There are other four genes that have been annotated as putative acidic endochitinase genes in the *Musa* genome project. The ‘novel’ endochitinase gene identified in this study encodes a 282-aa peptide, which shares a 77% sequence identity with another annotated acidic endochitinase (GSMUA_AchrUn_randomP12350_001) in a 177-aa region. Therefore, this ‘novel’ gene was apparently missed in the genome annotation process or due to incomplete genome assembly.

Aside from the ‘novel’ transcripts that show sequence similarity to other plant and/or non-plant genes, the remaining novel transcripts encode deduced peptides that share no sequence similarity to any other proteins at the E-value cutoff 1e-5. They are likely from banana-specific genes. Additional file 2: Table S2 lists 151 transcripts which are derived from these putative banana-specific genes. The list only includes the ones that have a minimal length of 259 nt and a minimal abundance of 0.56 FPKM by RNA-seq. Additional file 3: Figure S1A plots the distribution of length of these putative banana-specific transcripts and their encoded peptides. Among them, 15 transcripts contain a predicted ORF that encodes a peptide of at least 150 amino acids, but the predicted peptides encoded by the majority of these putative banana-specific transcripts are shorter, suggesting that many of them might be non-coding RNAs. Most of the 151 banana-specific transcripts were expressed with less than 5 FPKM, but 44 of them have a FPKM higher than 5 (Additional file 3: Figure S1B).

It needs to be noted that in addition to the novel transcripts listed in Additional file 1: Table S1, some of the other RNA-seq sequences that map to un-annotated genes could also be transcribed from genuine genes. All these assembled RNA-seq sequences are publically accessible through GenBank (Accession No. SRX317049).

### Identification of single nucleotide polymorphisms (SNPs) and short insertions/deletions (indels)

The genome of cultivated Cavendish-type banana is believed to be highly heterozygous as it was derived from an intra-species cross of *Musa acuminata*, a cross-pollinating species. The *Musa* genome sequence was obtained through sequencing the doubled-haploid *M. acuminata* genotype [6]. Therefore, allelic polymorphisms that exist in the cultivated triploid banana cultivars could not be revealed by the sequenced genome data alone. Identification of SNPs and indels will reveal allelic polymorphisms, useful information for breeding programs and for studying their origins. The transcriptome sequences from the Cavendish cultivar are a good source to identify such polymorphisms in genes.

Using SAMtools, a total of 21,451 SNPs and 3,207 indels were identified from our transcriptome data (Additional file 4: Table S3). We only listed the SNPs/indels that were identified by at least two sequence reads. If it was hit only by a single read, it is more likely from a sequencing error and therefore not regarded as a real SNP/indel in this report. Besides, we only examined SNPs/indels in the transcripts that map to the annotated banana genes or the 842 novel transcripts described earlier that have not been annotated in the genome.

The cultivated banana is a triploid; however, we did not find a polymorphic site that differs in all three different alleles. Commonly, for each polymorphic site, two alleles show the same sequence whereas the third allele shows an allelic variation. This result further indicates that one parent/ancestor of the triploid cultivated banana contributed two sets of chromosome whereas the other one contributed one set of chromosome. Allelic variations that lead to gene products with altered functions could be an advantage for plant fitness under certain environments.

### GFP as a marker for monitoring the infection process by *Fusarium oxysporum* f. sp *cubense* (Foc)

Race 1 of Foc (Foc1) was responsible for the widespread epidemics of Panama disease before 1960s. Since then, Cavendish cultivars have been the most widely used cultivars for banana production because of its resistance to Race 1. Tropical Race 4 (Foc TR4) is highly virulent on Cavendish and has been causing the outbreaks in many regions in world in recent decades. Foc is a soil-borne pathogen that invades xylem tissues of roots and spread through the vascular system of pseudostems, particularly through xylem vessels. However, the infection process is difficult to monitor and the first sign of disease symptoms (leaf discoloration and wilting) appear several weeks after infection. Little is known about the difference in the early infection processes between Race 1 and Race 4.

**Figure 1:**
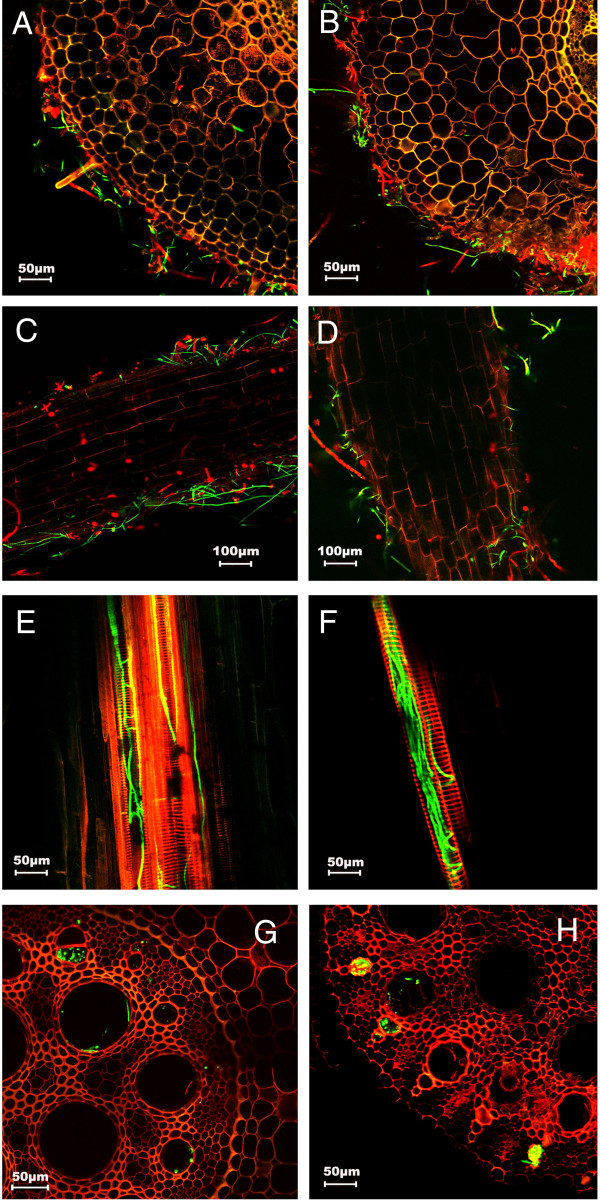
**Examination of the early infection process using GFP-expressing Foc1 and Foc TR4.** At 27 hrs post-inoculation, spores and hyphae of the GFP-expressing Foc 1 **(A and C)** and Foc TR4 **(B and D)** were attached to the roots. At 51 hrs post-inoculation, hyphae were observed in the vascular tissues 3-5 mm from the cut sites of the banana roots infected with Foc1 **(E and G)** and Foc TR4 **(F and H)**.

To provide assistance in monitoring the pathogen infection process, we transformed both Foc1 and Foc TR4 with the gene encoding a modified green fluorescence protein (sGFP) [10]. We selected the GFP strains of Foc1 and Foc TR4 which showed similar fluorescence intensity. The GFP-expressing strains were found to have similar morphological features and pathogenecity on banana plants to their wild strains. To monitor the infection processes, roots of banana plants were cut and inoculated with spores of GFP-expressing Foc1 and Foc TR4, and the fluorescence signal was observed under a confocal microscope. As shown in Figure 1, 27 hours (hrs) post-inoculation, spores and hyphae were attached to the banana roots inoculated with Foc1 (Figure 1A, 1C) or Foc TR4 (Figure 1B, 1D). At 51 hrs post-inoculation, hyphae spread into vascular tissues of the roots infected with Foc1 (Figure 1E, 1G) or Foc TR4 (Figure 1F, 1H). Our observation indicates that both Foc1 and Foc TR4 are capable of spreading into vascular tissues in roots at least during the first couple days of the infection process. Although it appeared that more hyphae were in the vascular tissues of the Foc TR4-infected roots than in the Foc1-infected roots, we could not find obvious difference in the early infection process by Foc1 and Foc TR4. However, it is difficult to make a precise quantitation of relative amount of hyphae using such a GFP reporter assay.

Although Cavendish cultivars are generally resistant to Foc1 strains, the mechanism of the resistance remain elusive. The sterile triploidy nature of these cultivars hampers determination of the genetic basis of the resistance trait. It is plausible that the Cavendish’s resistance to Race 1 is a polygenic quantitative trait as it is affected by various environmental factors. It has been reported that Foc1 can cause some degree of infection on Cavendish bananas under certain conditions although the severity of wilt disease is dependent on temperature, soil drainage conditions, soil pH, and inoculum levels [11–13]. Similarly, resistance to subtropical race 4 is also dependent on environmental conditions. For instance, VCG0120 of subtropical Race 4 can severely infect Cavendish bananas in the subtropical regions but not in the tropics [14]. We found a similar infection process by Foc1-GFP and Foc TR4-GFP in the first two days following the inoculation although the Foc1-GFP, like other Foc1 strains, did not eventually lead to obvious wilt disease in our laboratory or field conditions. The results suggest that the difference of Cavendish cultivars in resistance to Foc1 and Foc TR4 is largely due to a difference in later infection stages which could either be due to Foc TR4′s ability to overcome the host defense mechanism or the host’s ability in activating more effective defense mechanisms in response to Foc1 infection.

### Inoculation of banana plants by Foc1 and Foc TR4 for gene expression profiling analysis

To identify genes whose expression is altered in response to infection by Foc and to reveal any difference in global gene expression profiles following infection with Foc1 and Foc TR4, we cut root tips of banana seedlings and inoculated the wounded roots by immersing the roots to the Foc spore culture. The inoculated roots were harvested at 3 hrs, 27 hrs, and 51 hrs after the initial inoculation for RNA extraction. The plants whose roots were immersed in the culture medium without the pathogen (mock inoculation) were used as a control. The gene expression profiles at the 3 hrs time point is considered to reflect an early host response triggered mainly by pathogen-associated molecular patterns. The profiles at 27 hrs and 51 hrs time points can be regarded as an early-intermediate response to infection by the Foc strains. The three time points were designed in such a way that all tissue samples were collected at the same time in each of these three days to minimize differences in circadian-influenced gene expression when comparing their transcriptome profiles. The control samples were also collected at the same time points following mock-inoculation. RNA extracted from the roots was subjected to digital gene expression (DGE) analysis.

### Identification of DGE tags representing expressed genes

The sequence tags (Accession No. SRX317053) derived from the DGE sequencing libraries were mapped to the virtual tags *in silico* extracted from the annotated genes of the *Musa* genome and the novel transcripts from our RNA-seq results as well as to the full *Musa* genome sequence. The genuine sequence tags should be mapped to the virtual tags at the forward direction. However, some tags were mapped to the virtual tags at reverse direction or to the antisense strands. Others mapped to un-annotated genome regions or to positions beyond the *NlaIII* sites. Those tags that are not mapped to the virtual tags could be from unidentified genes or are anti-sense transcripts; however, they could also come from genomic DNA contamination or from sequencing errors or sequence assembly errors. In this report, further gene expression profiling analysis was focused on the sequence tags that are mapped to the virtual tags of the corresponding sequences in the annotated genome or to the transcripts identified based on our RNA-seq results. The counts of all the tags mapped to the same gene were added up and normalized by the total mapped reads in the library as TPM (transcripts per million).

**Figure 2:**
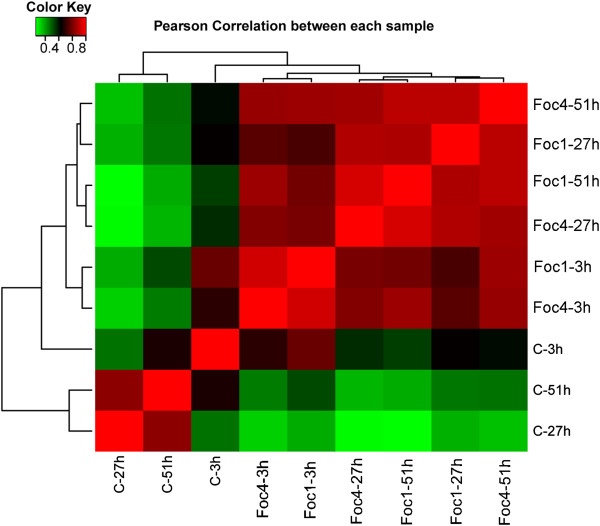
**Pearson correlation of the samples.** Each grid denotes the corresponding Pearson correlation coefficient between the two samples. C-3 h, C-27 h, and C-51 h are the control samples harvested at 3 hrs, 27 hrs and 51 hrs post mock-inoculation, respectively. The other samples were Foc1- or Foc TR4-inoculated samples harvested at the indicated time points.

Additional file 5: Table S4 lists all distinct transcripts identified by the DGE tags and their expression levels. Some of them were also detected as antisense transcripts. Among those transcripts, 434 transcripts (whose names are started with CUFF in the table) are from regions that were not annotated as genes in the genome project but were found from our RNA-seq transcriptome data (as described earlier). A total of 11412 banana transcripts were identified with greater than 3 TPM in at least one DGE sample, and most of them were low-abundant with 3-10 TPM. The expression abundance for each transcript in all libraries was used to calculate the Pearson correlation coefficients (Figure 2). Two of the mock-inoculated control samples, 27 hrs and 51 hrs post-mock-inoculation, have high correlation (0.83). However, the overall expression profile of the 3 hrs control sample was found to be more similar to the samples of 3 hrs post-inoculation with Foc1 or Foc TR4 than to the other two mock-inoculated control samples, presumably because these three 3 hrs-time point samples have similar expression patterns of many wounding-responsive genes (caused by cutting of the root tips for pathogen infection and mock inoculation). Besides, all 4 samples collected at 27 hrs and 51 hrs post-inoculation by Foc1 or Foc TR4 showed a high overall similarity (>0.88).

### Identification of Foc-responsive genes

**Figure 3:**
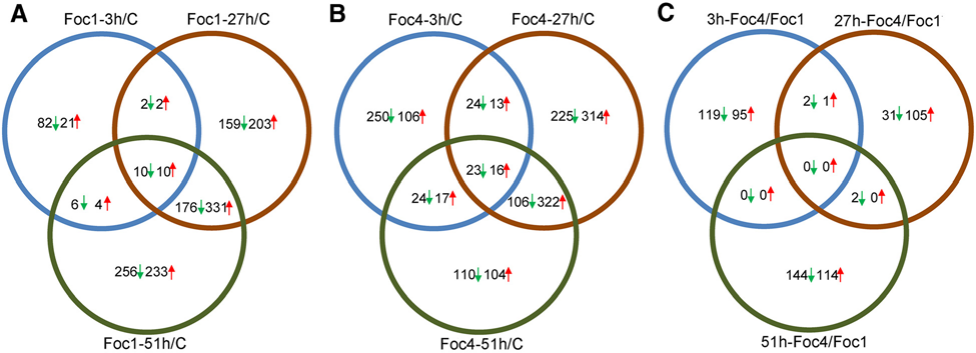
**Venn diagram comparison of differentially expressed genes. A-B**. The number of differentially expressed genes derived from comparison between the Foc1-infected **(A)** or Foc TR4-infected **(B)** samples and the control sample at each time point. Green arrows denote genes down-regulated in infected samples, while red ones are up-regulated genes. **C**. Comparison between Foc TR4-infected and Foc1-infected samples at each time point. Green arrows denote genes that were expressed at a lower level in the Foc TR4-infected sample than that in the Foc1-infected sample whereas the red arrows indicate genes expressed at a higher level in the Foc TR4-infected sample than the Foc1-infected sample.

We compared the transcript levels between pathogen-inoculated and corresponding mock-inoculated roots and between the roots inoculated with the different Foc races at 3, 27 and 51 hrs post-inoculation. Additional file 6: Table S5 lists differentially expressed genes with a fold change of 3.0 or higher in at least one of the nine comparisons. The numbers of the genes showing statistically significant changes were plotted in the Venn diagrams (Figure 3). Figure 3A-3B show comparison of the Foc-responsive genes at the different time points following inoculation with the same Foc race, whereas Figure 3C shows comparison of transcript levels caused by infection with the two different races at each of the three time points.

Overall, a small number of genes were found up- or down-regulated at 3 hrs post-inoculation (Figure 3A and 3B). In contrast, a much larger number of genes showed altered expression levels in Foc1- or Foc TR4-inoculated roots at the later infection stages (27 hrs and 51 hrs). For example, 893 and 1026 genes showed altered expression at 27 hrs and 51 hrs after Foc1 inoculation, respectively. Similarly, 722 and 1043 genes were found to be differentially expressed at 27 hrs and 51 hrs after Foc TR4 inoculation, respectively. Among the Foc1-responsive genes, 20 genes were found to have altered expression in all three time points, whereas among the Foc TR4-responsive genes, 39 of them showed alteration in all three time points. Overall, we found very similar global gene expression patterns influenced by both Foc1 and Foc TR4. A large number of genes were up- or down-regulated at both 27 hrs and 51 hrs post-infection by Foc1 or Foc TR4. However, the number of the genes up- or down-regulated by both Foc1 and Foc TR4 at all three time points (Figure 3C) was much smaller due to the small number of Foc-responsive genes at 3 hrs post-infection. Four genes were up-regulated and five genes were down-regulated at all three time points by both strains (Table 1). Table 2 lists the genes that showed at least 10 fold difference in their transcript levels between the Foc1- and Foc TR4-inoculated roots at one or more time point.

**Table 1:** **The genes that were up-/down-regulated by Foc1 and Foc TR4 at all three time points**

Gene ID	Functional annotation	Symbol	Foc1/mock			Foc TR4/mock		
			3 h	27 h	51 h	3 h	27 h	51 h
2G11090	Putative GDSL esterase/lipase	At1g71250	3.69	20.71	68.12	4.38	18.46	74.36
3G14470	Alpha-glucan water dikinase	R1	3.16	6.52	15.36	3.97	5.51	9.05
7G16510*	Thaumatin-like	tlp	10.05	13.30	16.94	8.34	28.30	19.32
7G16510	Thaumatin-like	tlp	8.23	12.67	16.49	5.25	29.57	20.91
8G14290	unknown	unknown	3.87	16.16	42.72	3.48	42.62	28.26
3G30560	Cytokinin-O-glucosyltransferase 1	UGT73C1	0.31	0.19	0.03	0.16	0.04	0.22
10G21100	unknown	Slc4a10	0.99	0.33	0.16	0.29	0.15	0.30
3G07350	Dihydrolipoyllysine-residue acetyltransferase component of pyruvate dehydrogenase complex	pdhC	0.84	0.18	0.07	0.17	0.18	0.16
4G18790	Sorting and assembly machinery component 50 homolog	samm50	0.82	0.16	0.16	0.26	0.24	0.25

*: antisense transcript.

**Table 2:** **The genes that showed differential expression to the different Foc races**

Gene ID	Functional annotation	Symbol	3 h	27 h	51 h
			Foc TR4 /Foc1	Foc TR4 /Foc1	Foc TR4/Foc1
1G10090	expressed protein	waaU	**10.41**	1.00	0.49
5G14840	Putative Importin-5	Ipo5	**11.59**	0.35	0.75
10G08810	Putative Nitrile-specifier protein 4	NSP4	0.57	**16.05**	0.21
10G01250	Lichenase	GN1	0.59	**20.36**	1.11
CUFF.13486.1	early nodulin 93		0.98	6.34	**0.10**
1G10380	xyloglucan endotransglucosylase/hydrolase protein 7	XTH7	0.41	0.60	**10.00**
2G10960	Defensin-like protein	PDF2.3	0.43	0.52	**10.12**
9G11920	Peroxidase 1	PRX74	1.11	0.81	**10.95**
3G29530	Beta-glucosidase 1	BGLU1	0.29	0.26	**11.69**
5G10940	Trypsin inhibitor	BBI	0.54	0.17	**19.53**
CUFF.21673.1	14 kda proline-rich protein		0.69	0.22	**34.74**
CUFF.10813.1	fasciclin-like, arabinogalactan protein 11-like		0.38	0.11	**38.77**
4G08950	Intracellular ribonuclease LX	RNALX	0.39	**0.08**	**15.39**

Note: The numbers in bold represent the changes that are 10 folds or higher.

**Figure 4:**
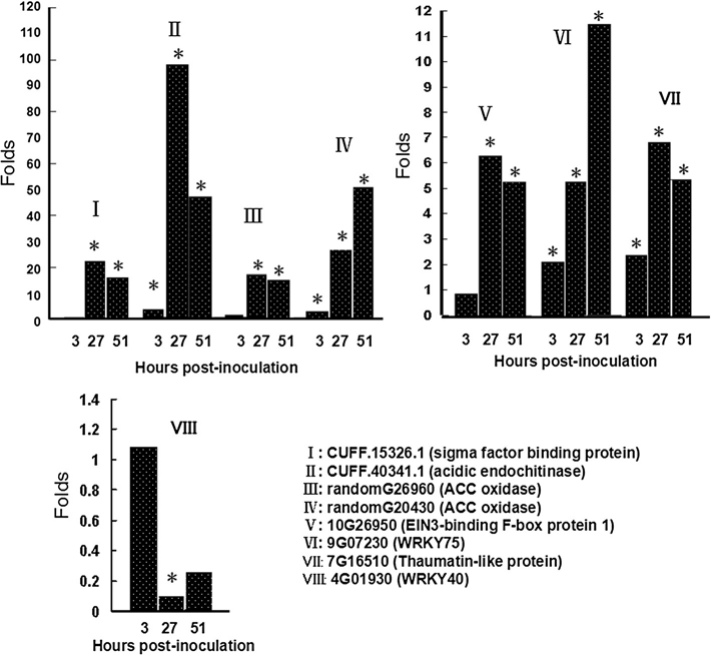
**Expression levels of the selected Foc-responsive genes determined by qPCR.** The bars represent the fold changes of the transcript levels based on comparison between Foc1-inoculated and mock-inoculated samples. The star (*) indicates that the difference between the infected and control samples is statistically significant (p < 0.05).

Several genes whose expression was found altered by Foc infection were chosen for real-time quantitative PCR (qPCR) analysis to compare their transcript levels between Foc-inoculated and mock-inoculated roots that were prepared independently from the DGE samples. Those genes are marked with a star symbol in Table 3 which lists a selected set of the Foc-responsive genes. Since the expression of these genes was largely similarly affected by Foc1 and Foc TR4, only Foc1-inoculated roots were collected for the qPCR analysis. Among the analyzed genes, the ones that showed a similar expression pattern revealed in the qPCR analysis and the DGE results include two ACC oxidase genes (randomG26960 and randomG20430, neither of which has been mapped to a particular *Musa* chromosome), a *SIB1*-like gene (CUFF.15326.1), a thaumatin-/*PR5*-like genes (7G16510), an *WRKY75*-like gene (9G07230), an acidic endochitinase gene (CUFF.40341), and a gene encoding a homolog of the EIN3-binding F-box protein 1 (Figure 4A). Based on the DGE result, the transcript encoding a homolog of the Arabidopsis WRKY40 (At4g01930) was found to be reduced by more than 10 folds at 3 hrs and 51 hrs post-infection with Foc1 compared with the mock-inoculated samples. This gene was found to show approximately 10 fold reduction at 27 hrs post-infection with Foc1 from the qPCR result; however, its transcript level was found to be reduced by approximately 3 folds (not statistically significant) at 51 hrs but was unchanged at 3 hrs post-infection based on the qPCR result (Figure 4B). Other two genes examined by qPCR did not show a similar expression pattern to that from the DGE results, which include a gene encoding a putative transcription factor (CUFF.14993) and the gene encoding a homolog of the Arabidopsis ethylene responsive transcription factor 2. The inconsistence between the DGE and qPCR results for some of these genes could be due to false positives/negatives resulted from either of these two methods or experimental variation caused by different batches of plants and pathogens or other unidentified factors.

**Table 3:** **A summary of selected Foc-responsive genes**

Gene ID	Foc1	Foc TR4	Foc1	Foc TR4	Foc1	Foc TR4	Gene annotation
	3 hrs		27 hrs		51 hrs		
**Pathogenesis-related and other known defense-related genes**							
7G16510*	8.2	5.2	12.7	29.6	16.5	20.9	Pathogenesis-related protein, thaumatin-like protein
6G31470	4.3	2.6	70.1	>100	41.0	46.6	Thaumatin-like protein
1G02580	0.6	0.2	10.2	4.7	10.2	15.0	Thaumatin-like protein
6G21330	1.1	0.7	5.4	11.4	10.7	25.1	Endochitinase
CUFF.40341.1*	6.3	2.1	2.3	3.9	6.8	65.9	Acidic endochitinase
CUFF.18858.1	4.8	0.1	0.2	0.7	0.2	0.3	Non-specific lipid-transfer protein 2-like
5G10940	0.6	0.3	0.1	0.0	0.0	0.1	Trypsin inhibitor
11G16380	1.6	0.9	>100	>100	45.5	40.6	Phenylalanine ammonia-lyase 3 (PAL3)
9G30640	3.6	1.4	89.8	>100	9.2	18.7	Germin family protein 12
randomG28090	0.9	0.3	8.8	10.5	4.5	23.8	Germin family protein 3
10G01250	7.5	4.5	27.8	>100	37.0	40.9	Lichenase (endo-(1-- > 4)-beta-glucanase)
9G23850	0.79	0.92	>100	>100	>100	>100	Lignin-forming anionic peroxidase
CUFF.1900	0.8	0.3	8.4	13.4	10.0	12.6	bon1-associated protein 2 (BAP2)-like
5G08810	1.0	0.2	0.1	0.2	0.1	0.4	Allene oxide synthase 2 (CYP74A2)
**Ethylene biosynthesis and signaling**							
randomG26960*	2.7	1.4	65.0	38.4	>100	>100	1-aminocyclopropane-1-carboxylate oxidase
randomG20430*	2	0.4	>100	>100	>100	>100	1-aminocyclopropane-1-carboxylate oxidase
5G09690	1.7	1.2	3.7	30.7	7.6	2.8	1-aminocyclopropane-1-carboxylate oxidase
2G07300	0.5	0.7	1.6	9.5	19.1	3.8	Putative Ethylene-responsive transcription factor 1 (ERF1)
4G05520	0.5	0.6	19.6	50.9	>100	>100	ERF1B
randomG27100	0.5	0.4	16.3	66.3	>100	83.0	ERF071
randomG16100	1.3	1.2	10.3	21.4	24.2	9.9	ERF9
11G20930	0.7	0.7	7.0	10.5	7.8	7.2	ERF2
**Transcription factors**							
2G20130	0.5	1.6	22.4	13.5	8.3	9.5	Putative WRKY transcription factor 6 (WRKY6)
4G01930*	0.0	0.1	0.7	0.8	0.1	0.1	WRKY40
randomG14780	2.0	10.4	>100	>100	35.3	37.2	Putative Transcription factor ICE1
5G14600	0.9	1.7	11.2	12.8	96.2	12.7	Putative Myb-related protein Myb4
5G01960	0.98	3.71	>100	>100	>100	>100	Homeotic protein knotted-1
CUFF.15326.1*	0.4	0.6	>100	>100	6.6	11.9	Sigma factor binding protein (SIB)1
**Kinases**							
9G19130	0.4	0.4	0.0	0.4	0.1	0.0	Mitogen-activated protein kinase kinase kinase ANP1
6G07600	0.6	1.7	62.1	65.7	94.5	67.7	STE_MEKK (mkkA)
10G21160	0.66	5.02	>100	>100	>100	>100	Mitogen-activated protein kinase kinase 2
10G07820	1.5	0.6	>100	>100	14.7	22.0	BRASSINOSTEROID INSENSITIVE 1-associated receptor kinase (BAK)1-like
2G02850	2.3	1.1	1.1	0.9	7.6	1.3	Putative Wall-associated receptor kinase (WAK 1)
6G15260	0.8	0.8	2.2	3.0	5.3	3.8	WAK2
4G09850	1.0	9.4	>100	40.9	5.4	5.7	WAK4

*Expression patterns of these genes were examined by qPCR.

### Functional categorization of a selected set of Foc-responsive genes

Table 3 contains the information of a list of selected genes whose transcript levels were altered by Foc infection based on the DGE results. Some of them are well-known defense-related genes. Two *PR5-like* (thaumatin-like) genes were found to be up-regulated by both strains at all three time points with the highest expression level at 1-2 days post-inoculation. Another thaumatin-like gene was induced only at the later (1-2 days) time points by both strains. A *PR4*-like (endochitinase) gene was also strongly induced (up to 25 folds) by both strains at the later time points. Another Foc-induced gene encodes a protein similar to bon1-associated proteins (BAP) in Arabidopsis. BAP1 and BAP2 are homologous proteins containing a calcium-dependent phospholipid-binding C2 domain and both function in the defense pathway [15]. A gene encoding a sigma factor binding protein (SIB) was also highly induced by both Foc strains at 1-2 days post-infection but not at the 3 hrs time point. SIB1 and SIB2 in Arabidopsis are positive regulators in defence against both biotrophic and necrotrophic pathogens and bind to and activate WRKY33 [16, 17]. Two WRKY genes are among the Foc-responsive genes. Many WRKY genes act as positive or negative regulators in various biotic and abiotic stress responses. A *WRKY6-like* gene was found induced by Foc at 1-2 days post-infection whereas the transcript level of a *WRKY40*-like gene was reduced following infection by Foc1 or Foc TR4. WRKY40 is a key negative regulator of the defense pathway, including the PAMP-mediated innate immunity [18, 19]. If the banana *WRKY40-like* gene has a similar function to that of the Arabidopsis *WRKY40*, its suppression by Foc is expected to enhance activation of the defense pathway.

The gene encoding BAK1 (BRASSINOSTEROID INSENSITIVE 1-associated receptor kinase 1) was highly induced in both Foc1 and Foc TR4-inoculated roots at 1-2 days post-inoculation. BAK1 is a receptor kinase and functions in both the brassinosteroid signalling pathway and the immune response [20, 21]. Brassinoteroids have been implicated in plant defence in dicot and monocot plants [22, 23]. In Arabidopsis, BRI1 recruits and phosphorylates BAK1 to initiate the BR signalling pathway [24]. Similarly, upon binding of bacterial flagillin to the receptor FLS2, FLS2 recruits BAK1 as a co-receptor to initiate the innate immune response [20]. BRs enhance the immune response when the BAK1 level is not rate limiting by supplying activated BAK1 for the defense pathway [25, 26]. The increasing BAK1 level in the Foc-treated banana roots might potentiate the innate immune response. However, it remains to be determined whether BAK1 is also employed in defense against fungal pathogens.

A gene encoding phenylalanine ammonia-lyase and another one encoding lignin-forming anionic peroxidase were similarly induced (over 40 folds) by both Foc1 and Foc TR4 at 1-2 days post-infection. PALs are involved in biosynthesis of phenolpropanoids, monolignols, and phytoalexins. Monolignols can be polymerized by peroxidises to form lignins, which could fortify the cell walls. A gene encoding a lignin-forming anionic peroxidase is strongly induced by both Foc 1 and Foc TR4 at 27 hrs and 51 hrs post-infection.

Two genes encoding germin-like protein are among the strongly Foc-induced genes, particularly at 1-2 days post-inoculation. One of these two germin genes was also induced (over 4 folds) at 3 hrs post-infection by Foc1 but not by Foc TR4. Some germin family proteins are oxalate oxidases which are involved in production of reactive oxygen species and are known to function in biotic and abiotic stress responses [27].

Interestingly, several genes involved in ethylene biosynthesis and regulation of ethylene-responsive genes were induced by the infection, particularly at 1-2 days post-inoculation. Three genes encoding 1-aminocyclopropane-1-carboxylate oxidase (ACC oxidase) are among the Foc-responsive genes. ACC oxidase catalyzes the last step of ethylene biosynthesis by converting ACC to ethylene [28]. These three ACC oxidase genes were slightly induced (1.7-2.7 folds) at 3 hrs post-inoculation with Foc1. Besides, 5 genes encoding ethylene-responsive transcription factors (ERF) were also strongly induced 1-2 days post-infection with both strains although the result for one of them (*EFR2-like*) could not be confirmed by qPCR.

Pathogen infection, particularly by necrotrophic pathogens, often triggers accumulation of jasmonate (JA) which acts as a key signaling molecule in regulation of the plant defense pathways [29]. JA also has an antagonistic effect on the SA-mediated signaling pathway which is activated by biotrophic pathogens. The first committed step of jasmonate synthesis from free fatty acids is catalyzed by allene oxide synthase [30]. We found that a gene encoding allene oxide synthase 2-like protein was suppressed in the Foc-treated roots, particularly at 1-2 days post-inoculation by both Foc strains. At 3 hrs post-infection, only the Foc TR4-inoculated roots, but not the Foc1-inoculated roots, showed suppression of the allene oxide synthase gene. The result suggests that Foc infection might lead to reduction of JA although it remains to be determined whether this allene oxide synthase gene is indeed responsible for JA production in banana in response to Foc infection.

DGE-based gene expression profiling studies in banana roots infected with Foc TR4 has previously been reported [31, 32]. Our results on expression patterns of some Foc-responsive genes are consistent with the previous reports but are different for some other genes [31, 32]. For instance, several genes involved in phenolproponoid biosynthesis were previously found to be induced by Foc TR4 [32]. *BAK1* was also found to be induced by Foc TR4 infection in banana by another report [33]. However, some jasmonate biosynthetic genes [32] and a JA signaling gene [34] were found to be induced by Foc TR4. We did not find significant induction of jasmonate biosynthetic-related genes but instead found suppression of the allene oxide synthase gene. Similarly, Wang et al [32] did not find induction of any ethylene biosynthetic or signaling pathway genes whereas Li et al [31] showed induction of *EIN3* by Foc TR4. Some of the differences could be due to the different experimental designs used for comparing gene expression levels. For instance, in the study by Wang et al [32], gene expression levels in the roots harvested at different time points following the infection were compared with the roots harvested before the infection. However, in our study, transcriptomes in the infected roots were compared with the mock-inoculated roots harvested at the same time points. Surprisingly, neither the previous reports nor this study found obvious induction of SA-responsive genes. The level of a *PR1-*like gene, one of well-known SA-responsive genes in many plant species, did not show change in its transcript level in our study either. Similarly, few JA-responsive genes were found to be induced by Foc infection. These studies suggest that the SA and JA signaling pathways might not be significantly activated during the early infection processes by either Foc1 or Foc TR4.

*F. oxysporum* infects a wide range of plant species including many economically important crops such as tomato, cotton, cabbages, legumes, and cucurbits. Plants evolved various mechanisms to defend against *F. oxysporum*[35]. The best studied examples are the interactions between tomato and *F. oxysporum* f. sp. *lycopersici* (Fol). Conventional breeding has been very successful in controlling tomato fusarium wilt largely through introgression of gene-for-gene mediated resistance [36]. Three resistance (R) genes (*I*, *I-2*, *I-3*) from wild tomato have been introgressed into cultivated tomato. Like a large majority of R genes, these three *I* genes encode the nucleotide binding site-leucine-rich repeat (NB-LRR) class proteins that recognize corresponding effector proteins secreted by specific Fol races to activate the defense response [36]. However, the I-mediated resistance apparently varies from the classical R-effector-mediated hypersensitive response that often leads to programmed death of infected cells. Instead, the I-activated response mainly involves callose deposition, phenolics accumulation, and formation of gels in the infected vessels which likely lead to vessel occlusion to prevent pathogen spreading [36]. The Foc infection-triggered induction of the banana genes involved in synthesis of phenolpropanoids (such as *PAL*) and cell wall strengthening (such as the lignin-forming anionic peroxidase gene) could also provide such a defense mechanism.

The effector proteins that are secreted from Fol and recognized by these I proteins are among so-called SIX proteins (Secreted In Xylem) [37, 38]. Some of the SIXs have been found to suppress the host’s basal defense and/or gene-for-gene resistance [reviewed in [36]]. The genome sequence of a Foc TR4 strain is now publicly available (http://www.broadinstitute.org/annotation/genome/fusarium_graminearum/MultiDownloads.html) and the genomes of another Foc TR4 strain and a Foc1 strain will likely be available soon (https://pag.confex.com/pag/xxi/webprogram/Paper6139.html). Comparison of the genomes of these two Foc races could lead to identification of SIX-like candidates which might contribute to the difference in their virulence to banana, and their functions in Foc’s pathogenecity can then be experimentally tested.

The sterile nature of triploid banana cultivars is a hurdle in determining genetic basis of their resistance/susceptibility to Foc. *M. accuminata* ssp. *malaccensis*, which is a wild diploid subspecies of the cultivated banana ancestor *M. accuminata*, has been found to be highly resistant to Foc4 and the resistance is controlled by a single dominant gene [39], which could be an *I-like* gene. Isolation of such a Foc resistance gene will be greatly helpful in using genetic transformation to improve banana cultivars’ resistance to Foc4.

R gene-mediated race-specific monogenic resistance is often evolutionally unstable because of evolving of new virulent pathogen races. In Arabidopsis, resistance to *F. oxysporum* f.sp. *matthioli* (Fom) is a polygenic trait controlled by at least 6 quantitative trait loci (QTL) termed *RESISTANCE TO F. OXYSPORUM* loci (RFO) [40]. Three RFO genes (RFO1-3) have been cloned and they belong to the receptor-like kinase (RLK) family [40–42]. RFO1 is a member of the wall-associated kinase (WAK) subfamily. At least three *WAK*-like genes were induced by the Foc infection (Table 3). Among them, *WAK2* and *WAK4* were induced by both Foc1 and Foc TR4 at the 27 hrs and 51 hrs post-infection whereas *WAK1* was induced only at 51 hrs post-infection by Foc1.

Ethylene is an important modulator in plant disease resistance; however, it differentially affects resistance against different types of pathogens [43]. Generally, the ethylene signaling pathway plays a positive role in resistance to necrotrophic pathogens such as *F. oxysporum* [43]. Overexpression of ERF1 in Arabidopsis, a transcription factor that activates ethylene-responsive genes, enhances resistance to *F. oxysporum* f. sp. *conglutinans* and f. sp. *lycopersici* [44]*.* Mutations that lead to ethylene insensitivity (such as *etr1-1, ein2*) make tobacco and Arabidopsis more susceptible to several formae speciales of *F. oxysporum* [44, 45]. Those studies demonstrate that the ethylene signaling pathway is important for resistance to fusarium wilt in those plants. In our study, several ERF-like genes were found to be strongly induced in 1-2 days following infection by both Foc1 and Foc4 TR4 (Table 3), which could enhance the ethylene response pathway.

Because of the difficulty in using conventional breeding for banana improvement, molecular biotechnology offers great hope for improving banana for enhanced disease resistance and for other agronomically important traits by overcoming the constrains imposed by the sterility of cultivated banana. A gene conferring a dominant resistance trait, such as a banana *I*-like R gene that can activate banana resistance against Foc TR4 or other genes that confer a broad spectrum resistance will be particularly useful in genetic engineering of banana for enhanced resistance. The availability of the genome sequences of banana and different Foc races, as well as the transcriptomes and global gene expression profiles, are very useful in future studies toward understanding the molecular mechanism mediating resistance to fusarium wilt disease.

## Conclusion

1. Through the analysis of the transcriptome data obtained through RNA-seq, we identified at least 842 putative banana genes that have not previously been annotated. The analysis also led to the identification of a large number of SNPs and indels in the banana genes.
2. No obvious difference was found in the early infection process (the first 2 days) between Foc1- and Foc TR4-infected roots, and both races were found to invade vascular tissues of banana roots. The global gene expression patterns influenced by infection of Foc1 and Foc TR4 were also highly similar. The results indicate that the difference in the Cavendish cultivar’s susceptibility to these two races might be due to host’s responses during later infection processes.
3. Foc infection induced expression of many genes commonly responsive to infection by other pathogenic microorganisms, including *PR* genes (such as thaumatin-like genes), the genes involved in synthesis of phytoalexins and phenolpropanoids (*PAL*) and cell wall strengthening (the gene encoding lignin-forming anionic peroxidase).
4. Several genes involved in ethylene biosynthesis and signaling pathways are among the strongly induced genes by Foc infection, suggesting involvement of this hormone in the banana response to the infection.

## Methods

### Plant materials

The banana cultivar (Baxi) used in this study is the Cavendish subgroup with the*Musa* AAA genome. Banana plantlets were propagated under a sterile tissue culture condition. Suckers were used for multiplication and rooting by placing in plastic bags containing a growth medium. The medium for subculturing contains 1x Murashige & Skoog (MS) basal salt mixture, 3% sucrose, 7% agar, 4.0 mg L^-1^ 6-benzylaminopurine, 0.5 mgL^-1^ α-naphthlcetic acid, pH5.8. The rooting medium is the same as above except with 2.0 mg L^-1^ 6-benzylaminopurine and 2.0 mgL^-1^α-naphthlcetic acid. The plantlets were grown in a 28°C growth room with a 16 h/8 h light/dark period and a light intensity of 5000 lux. Plantlets in the sealed bags were transferred to a greenhouse for 3-5 days and then removed from the bags and grown hydroponically for 50 days in the medium containing ¼ MS salts. Leaves, pseudostems, and roots were collected from those hydroponically grown plants for RNA extraction. Floral tissues and banana fruits at various developmental stages were collected in November, 2010 from a banana plantation field in Haikou, China. The tissues were frozen in liquid nitrogen and stored in -80°C freezers till use.

### RNA extraction

Total RNA was extracted from roots, pseudostems, leaves, floral organs, and developing fruits separately using a modified CTAB method briefly described below. Two to five grams of tissues were grounded in liquid nitrogen, and the powder was mixed with 20 mL CTAB buffer (3% CTAB, 2% PVP, 2.0 M NaCl, 20 mM EDTA, 100 mM Tris pH 8.0, 2% β- mercaptoethanol) and incubated at 65°C for 20 min. The extract was mixed with 0.6 volume of chloroform by vortexing and span at 12000 g for 15 min at room temperature. The supernatant was transferred to a new tube and extracted with an equal volume of chloroform, and the supernatant was then mixed with 0.5 volume of 12 M LiCl and incubated at -20°C for 2 hours. RNA was precipitated by centrifugation at 12000 g for 15 min at 4°C and the pellet was re-suspended in 1 mL 0.2 M NaCl. The RNA solution was extracted sequentially with an equal volume of water-saturated phenol (pH 4.2) and chloroform. RNA was precipitated by mixing the solution with three volumes of ethanol and leaving on ice for 30 min before centrifugation at 14000 g for 20 min at 4°C. After washing the pellet with 75% ethanol, the RNA pellet was dissolved in 50 μL RNase-free water. The quality of the RNA samples was checked by using Agilent 2100 Bioanalyzer. The sample for RNA sequencing was derived from pooling of the RNA samples isolated from the different tissues according to the following ratios: 2 roots:1 pseudostems:1 leaves:1 fruits:1 flowers.

### RNA processing for transcriptome sequencing

Poly(A)-enriched mRNA was purified from the total RNA samples using Sera-mega Oligo(dT) beads (Illumina) and fragmented with divalent cations at elevated temperature. The RNA fragments were used for cDNA synthesis by using the SuperScript cDNA synthesis kit (Invitrogen) with random hexamer primers (N6). After end repairing, cDNA fragments were ligated to adaptors, purified and PCR amplified to make the library which was then sequenced using Illumina HiSeq™ 2000.

### RNA processing for digital gene expression (DGE) analysis

The tag libraries were prepared using the NlaIII sample prep kit (Illumina) according to the manufacturer’s instruction. Following mRNA enrichment and cDNA synthesis as described above, 5′ ends of tags were generated by digesting with *Nla*III,. The fragments apart from the 3′ cDNA fragments connected to Oligo(dT) beads were washed away and the Illumina adaptor 1 was ligated to the sticky 5′ end of the digested bead-bound cDNA fragments. of the DNA fragments were cut with *Mme*I. After removing 3′ fragments with magnetic beads precipitation, Illumina adaptor 2 was ligated to the 3′ ends of tags. The adaptor-ligated cDNA tags were enriched by 15 cycles of linear PCR amplification and the resulting 85-bp fragments were purified from 6% acrylamide gel. After denaturing, the single-chain molecules were fixed onto the Illumina Sequencing Chip (flowcell) for sequencing.

### Transcriptome assembly and analysis from RNA-seq

The raw reads were cleaned by removing adaptor sequences and low quality reads with ambiguous ‘N’. TopHat, a splice junction mapper for RNA-Seq reads [7], was used to align RNA-seq reads to the *Musa* genome sequence (http://banana-genome.cirad.fr) with default parameters (minimal intron length 50 and maximum intron length 500000). Cufflinks [8] was then used to assemble the transcripts from the TopHat alignment results. Novel genes were identified by comparing all the assembled transcripts to banana genome annotation (http://banana-genome.cirad.fr/) by Cuffcompare in the cufflinks package. The novel loci found by Cufflinks were scanned for ORF (open reading frame) by coding annotation tool in Trinity package [46]. Those transcripts with a putative complete ORF were aligned to the NCBI nr (non-redundant) database and the UniProt plant protein sequences (http://www.uniprot.org/uniprot/?query=taxonomy%3a33090&force=yes&format=fasta) [9] by BLASTx to find homologous proteins. The transcripts with more than one exon or single exon but having hits to known proteins at E-value cutoff 1e-5 were reported as final novel transcripts although some of the other sequences could also derived from genes that have not been annotated.

### Identification of SNPs and indels

SAMtools [47] was used to analyze the possible SNPs and indels in the banana genome based on the transcriptome data. The original reads were mapped back to the assembled banana transcripts. The SNPs and indels were called using the mpileup tool in SAMtools package. The coverage of SNP/indel-matched reads was set as not smaller than 2. If a SNP/indel was identified only from a single read, it was considered to be likely from a sequencing error and therefore not regarded as a real SNP/indel in this study. To test the accuracy of SNP calling, we developed a statistical method to model the sequencing error distribution. The model is described briefly below. According to the Illumina Solexa sequencing technology report, the sequencing error rate should be lower than 2%, and accordingly, a relatively strict sequencing error rate, 0.02, was selected. Given the total read coverage of a nucleotide site (*N*) and the substitution coverage (*k*), the probability of a nucleotide in a specified site being caused by sequencing errors, *p*(x|x ≥ *k*), could be simulated as a Poisson distribution, with the single parameter λ (λ = 0.02*N). A nucleotide with a probability lower than the pre-defined significant level (0.05) should be considered as a potential SNP rather than a sequencing error. The *p* values of potential SNPs were further corrected with False Discovery Rate (FDR) for multiple statistical tests. Only those with corrected *p* values (*q* values) lower than 0.05 were considered to be real SNPs. More than 95% the SNPs detected with the above-described simplified SAMtools-based method showed *q* values lower than 0.05.

### Digital gene expression (DGE) data processing, virtual tag extraction, and mapping the DGE sequence tags

The adapter sequences “(N_10_)TCGTATGCCGTCTTCTGCTTG” were cut from the raw reads using FASTX-Toolkit (Version 0.0.13) (http://hannonlab.cshl.edu/fastx_toolkit/). The remaining tags were 17-18 nucleotides long. Each tag was further counted by a custom perl script.

Virtual tags from the annotated banana transcriptome, novel transcripts found from our own RNA-seq results, and the *Musa* genome sequence were extracted from both up- and down-stream sequences of all *NlaIII* (CATG) restriction sites. The downstream tags (25 nt long including “CATG”) were directly cut and marked as the sense strand, while the reverse complementary upstream tags were cut and marked as antisense strand. The predicted tags were named as “cds.tag”, “novel.tag”, and “genome.tag”, respectively, according to the reference sequences mentioned above.

The processed unique sequence tags were mapped to “cds.tag” first by BLAST (version 2.26) with the word length 17. The unmapped tags were gathered and further mapped to the full *Musa* cds (coding sequence) sequences. The remaining unmapped tags were mapped to “novel.tag”, the novel transcripts, “genome.tag”, and full genome sequences sequentially.

### Statistical analysis

The Bioconductor package DESeq [48] was used to normalize tag counts and obtain variance-stabilized expression values for each gene. Pearson correlation coefficients were calculated to examine the gene expression data across all the samples using R (http://www.r-project.org/). We used ‘heatmap.2′ function of the ‘gplots’ package in R to construct heatmaps of correlation coefficients for all 9 samples [49].

To eliminate background noise, the transcript abundance was set to 20 if the normalized value was below 20 when calculating fold change for comparison.

### Fusarium strains and generation of the GFP lines

The strains of *Fusarium oxysporum* f. sp. *cubense* (Foc) used in this study are the Tropical Race 4 (Foc TR4) VCG01213/16 and Race 1 (Foc1) VCG 0123 isolated from the Hainan island of China by Dr. Junsheng Huang (Environment and Plant Protection Institute, Chinese Academy of Tropical Agricultural Sciences, Danzhou, China). These strains were transformed with the vector pCT74 which carries a modified GFP (sGFP) [10]. Protoplasts of Foc TR4 and Foc1 were transformed using a polyethylene glycol/CaCl_2_-mediated transformation method as described previously [50]. Growth characteristics and pathogenicity of the GFP-transformed lines were examined using the inoculation procedures described previously [34]. The GFP-expressing Foc TR4 and Foc1 with the similar growth characteristics and virulence to the wild strains were used for this study. For the digital gene expression experiment, only the normal strains were used to inoculate banana roots.

### Pathogen preparation, inoculation, and microscopic observation of the infection process

The GFP-expressing strains were used to observe the infection process. A small block of Foc culture on an agar plate was added to the potato dextrose broth (PDB) liquid medium and grown at 28°C for 48 hours in a shaker rotating at 180 rpm. The number of spores in the culture was counted and PDB was added to a final concentration of 10^6^ spores/mL. Roots of banana plants grown hydroponically for 50 days (as described above) were cut at approximately 0.5-1 cm from the root tips, dipped into the Foc spore solution, and inoculated for 2.5 hours. For the control plants, their roots were dipped into PDB as mock-inoculation). The plants were then placed back to the normal hydroponic condition for the indicated time. The inoculated banana plants were examined daily following inoculation. For the microscopic examination, banana roots were prepared by first washing the roots in sterile distilled water before observation under a Laser Confocal Microscope (OLYMPUS, FV10-ASW) equipped with the filter blocks with spectral properties matching those of the GFP (488 nm) and root auto-fluorescence (543 nm and 595 nm).

To prepare tissue samples for extracting RNA for the gene expression profiling analysis, Foc TR4 and Foc1 cultures were used for inoculating banana roots as described above. At 3 hours, 27 hours and 51 hours post-inoculation (starting from the initial inoculation time), the roots of five to six banana plantlets subjected to the same treatment were pooled together and frozen immediately in liquid nitrogen for RNA extraction.

### Real-time quantitative PCR (qPCR) for determination of transcript levels

Total RNA was extracted from Foc1-inoculated and mock-inoculated roots as described above. First-strand cDNA synthesis was performed with 1.5 ug total RNA using the RevertAid first strand cDNA synthesis kit according to the manufacturer’s instruction (FERMENTAS, Shenzhen, China). Transcript levels were analyzed by real-time PCR using the SYBR Green PCR master mix (Applied Biosystems, Shanghai) and a StepOne Real-Time PCR System according to the manufacturer’s manual. Gene-specific primers were designed based on the sequence information of their 3′ untranslated regions (UTRs) (if known), whereas for the three genes lacking 3′ UTR information, the primers were designed by annealing to their unique coding regions. A banana actin gene (GenBank# HQ853237.1) and an ubiquitin gene (GSMUA_Achr5P00760_001) which were found to have relatively constant expression levels in all DGE samples were used as a standard for the qPCR analysis. The PCR reaction involved the following steps: 95°C for 30 s followed by 40 cycles at 95°C for 5 s and 60°C for 20 s. Three biological replicates were included in the qPCR assay. Statistical significance in the transcript level comparison between Foc1-infected and mock-infected samples were calculated using Student’s t test.

### Availability of supporting data

The raw reads of our RNA-seq and DGE data were deposited in the Sequence Read Archive under accession numbers SRX317049 (http://www.ncbi.nlm.nih.gov/sra/SRX317049) and SRX317053 (http://www.ncbi.nlm.nih.gov/sra/SRX317053).

## Electronic supplementary material

Additional file 1: Table S1: A list of 842 novel genes identified from analysis of the transcriptome data. (XLSX 970 KB)

Additional file 2: Table S2: A list of 151 putative novel banana-specific genes. (XLSX 116 KB)

Additional file 3: Figure S1: Overview of the putative novel banana genes identified from the transcriptome data. **Figure S1A.** Distribution of their transcript length and peptide length. **Figure S1B.** Their transcript length and abundance detected by RNA-seq. (TIFF 467 KB)

Additional file 4: Table S3: SNPs and indels identified through analysis of the banana transcriptome. (XLS 5 MB)

Additional file 5: Table S4: A list of all genes identified from the DGE tags. (XLSX 2 MB)

Additional file 6: Table S5: A list of all differentially expressed genes. (XLSX 210 KB)
