# Supplementary figures and images for: Analysis of banana transcriptome and global gene expression profiles in banana roots in response to infection by race 1 and tropical race 4 of Fusarium oxysporum f. sp. cubense
Source: BMC Genomics. 2013 Dec 5;14(1):851. doi: 10.1186/1471-2164-14-851 (PMC4046742; doi:10.1186/1471-2164-14-851)

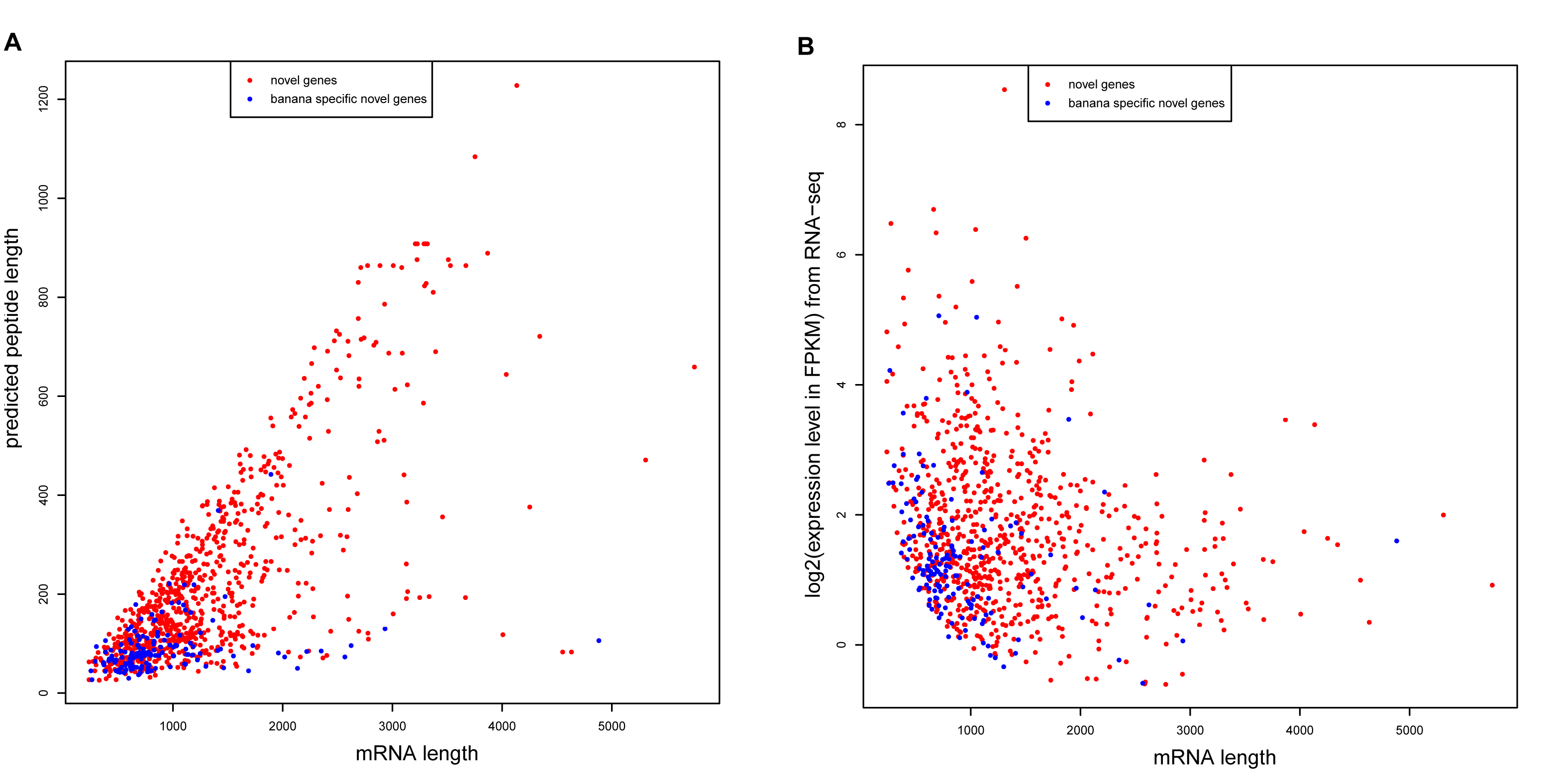

Supplement: Supplementary file 3 — Additional file 3: Figure S1: Overview of the putative novel banana genes identified from the transcriptome data. Figure S1A. Distribution of their transcript length and peptide length. Figure S1B. Their transcript length and abundance detected by RNA-seq. (TIFF 467 KB) [file 12864_2013_5566_MOESM3_ESM.tiff]
